# Transcriptomic landscape of the interaction between the entomopathogenic fungus *Beauveria bassiana* and its tolerant host *Tribolium castaneum* revealed by dual RNA-seq

**DOI:** 10.1038/s41598-023-43889-y

**Published:** 2023-10-02

**Authors:** María Constanza Mannino, Belén Davyt-Colo, Carla Huarte-Bonnet, Luis Diambra, Nicolás Pedrini

**Affiliations:** 1 Instituto de Investigaciones Bioquímicas de La Plata (INIBIOLP), CCT La Plata Consejo Nacional de Investigaciones Científicas y Técnicas (CONICET), Universidad Nacional de La Plata (UNLP), Calles 60 y 120, 1900 La Plata, Argentina; 2 Centro Regional de Estudios Genómicos (CREG), Facultad de Ciencias Exactas, Universidad Nacional de La Plata (UNLP), Boulevard 120 1459, 1900 La Plata, Argentina; 3 CONICET, La Plata, Argentina

**Keywords:** Bioinformatics, Gene regulatory networks

## Abstract

Entomopathogenic fungi such as *Beauveria bassiana* are the only insect pathogens able to start the infection process by penetrating through the host cuticle. However, some insects try to avoid fungal infection by embedding their cuticle with antifungal compounds. This is the case of the red flour beetle *Tribolium castaneum*, which generates economical loss of great significance in stored product environments worldwide. In this study, *T. castaneum* adults were fed during different time periods (from 3 to 72 h) on *B. bassiana* conidia-covered corn kernels. The progression of fungal infection was monitored using the dual RNA-seq technique to reconstruct the temporal transcriptomic profile and to perform gene enrichment analyses in both interacting organisms. After mapping the total reads with the *B. bassiana* genome, 904 genes were identified during this process. The more expressed fungal genes were related to carbon catabolite repression, cation binding, peptidase inhibition, redox processes, and stress response. Several immune-related genes from Toll, IMD, and JNK pathways, as well as genes related to chitin modification, were found to be differentially expressed in fungus-exposed *T. castaneum*. This study represents the first dual transcriptomic approach to help understand the interaction between the entomopathogenic fungus *B. bassiana* and its tolerant host *T. castaneum*.

## Introduction

Insect-pathogenic microbes are key components of integrated pest management strategies worldwide. They are extensively used for the control of insects such as pests of crops, forests, urban habitats, and arthropods of medical and veterinary importance^1^. Unlike other insect pathogens such as protozoa, bacteria, and viruses, entomopathogenic fungi can infect their host by adhesion to the surface and penetration through the cuticle^2^. However, some insects have evolved by embedding their cuticle with antifungal compounds, and thus become recalcitrant to entomopathogenic fungal infections^3^. In this sense, the family Pentatomidae produce saturated and α,β-unsaturated aldehydes^4^ and many species belonging to the family Tenebrionidae secrete methyl- and ethyl-benzoquinones^5^. All these carbonyl-based functional group compounds are responsible for the inhibition of both fungal germination and growth on the cuticle in the bed bug *Cimex lectularius*^6^, the stink bugs *Nezara viridula*^7^ and *Tibraca limbativentris*^8^, as well as the red flour beetle *Tribolium castaneum*^9^.

The red flour beetle is a major pest in structures used for the processing and storage of grain-based products, representing a serious threat for developing countries where its action results in significant agricultural losses per year^10^. Many studies have shown that adult *T. castaneum* is little susceptible to different isolates of the entomopathogenic fungus *Beauveria bassiana* via cuticular penetration, even after immersion at high doses of conidia^11–13^. Thus, the possibility of infection combining two routes of entry (i.e., the canonical cuticular route and the ingestion of conidia added to the insect's diet) represents an alternative that is worth exploring in this species, since it can mean an opportunity to increase effectiveness against the red flour beetle but other fungus-recalcitrant insects as well.

Dual RNA sequencing is a powerful transcriptomic technique to study host–pathogen interactions^14^. It offers an unbiased and global understanding of the transcriptomes of both host and pathogen (if their whole genomes are available) and have provided new insights into pathogenesis by identifying new virulence factors in the pathogen, mostly bacteria, or new pathways in the host cell that respond to the exposure to specific pathogens^15^. In fungus-caused invertebrate pathologies, dual RNA-seq was first applied to study the interaction between *B. bassiana* and the diamond-back moth^16^, and then used to study the global gene expression in other fungus-insect interacting systems^17,18^.

This study represents the first dual transcriptomic approach to help understand the interaction between the entomopathogenic fungus *B. bassiana* and its tolerant host *T. castaneum*. Insects were fed on conidia-covered corn kernels during different time periods (from 3 to 72 h), and the temporal transcriptomic profile, gene ontologies, and gene enrichment analyses were reconstructed by dual RNA-seq in both interacting organisms. A total of 904 fungal genes were found to be expressed during this process. After identification by mapping with the fungal genome, the more expressed genes were related to carbon catabolite repression, cation binding, peptidase inhibition, redox processes, and stress response. In *T. castaneum*, some genes related to chitin modification, and immune response related to Toll, IMD, and JNK pathways were differentially expressed as the fungal infection progresses.

## Methods

### Fungi

The entomopathogenic fungus *B. bassiana* strain ARSEF 2860 (ARSEF is WDCM collection number 112) was selected for this study since its whole genome is available^19^. To obtain conidia, the fungus was maintained and routinely grown at 26 °C for 15 days in Potato Dextrose Agar (PDA) medium with 1% ampicillin plates. Harvested conidia were suspended in 0.01% Tween 80 sterile solution, vortexed, and the concentration was adjusted to 1 × 10^9^ conidia/ml.

### Insects

Adult individuals of *T. castaneum* from colonies maintained at the Instituto de Investigaciones Bioquímicas de La Plata (INIBIOLP) were used. Insects were reared on white wheat flour, with 5% non-fat dried milk, 5% brewer’s yeast and 5% wheat germ, under a 12L:12D photoperiod at 27 ± 2 °C and 70 ± 5% relative humidity. All adults used in the assays were 2-week-old, unsexed insects.

### Insect exposure to fungi

A mixture of a conidial suspension (1 × 10^9^ conidia/ml) and wheat flour was optimized to cover a corn kernel with a thin layer. The mixture was prepared with 5 ml of conidial suspension and 7.5 g of wheat flour. Control kernels were similarly prepared but mixing wheat flour with 0.01% Tween 80 sterile solution instead the fungal suspension. Kernels were dried at 4 °C for 24 h. One kernel, either fungus-covered or control, was put inside separated Petri dishes and offered to 10 beetles. Insects were maintained feeding the kernel for different time periods from 3 to 72 h, then separated from the feeding arena and maintained in rearing conditions either for mortality bioassays or used for RNA extraction. Insects separated from kernels were sequentially rinsed in NaClO (10%) for 15 s, ethanol (70%) for 15 s and sterile Tween 80 (0.01%) for 2 min and dried in sterile absorbent paper for downstream treatments.

### Mortality and colony formation units (CFU) bioassays

Mortalities of *T. castaneum* fed during 3, 6, 12, 24, 48 and 72 h either on a conidia-added diet or on control kernels were checked daily, and cadavers were separated after each count. Mortality data were corrected for control mortality using the Abbott’s equation^20^. Ten replicates containing ten insects each were done.

For CFU assay, insects were first washed as described above to discard any conidia attached to the surface, and then carefully dissected to separate the heads from the rest of the body. To obtain the conidia inside insects, each part was vortexed in 0.01% Tween 80 sterile solution for 1 min. Serial dilutions of the vortexed solution were plated in PDA, incubated at 26 °C for 5 days and checked daily for colony formation. Heads and bodies were also incubated for 7 days and checked for fungal growth under the described conditions. Ten replicates containing ten adult beetles were used for each condition. Statistical analysis was performed using two-way analysis of variance (ANOVA) followed by the Bonferroni’s posttest (*p* < 0.05) using GraphPad Prism 8 (GraphPad Software Inc., USA).

### Total RNA extraction

Total RNA (fungus + insect) was extracted from pools of ten insects fed a conidia-added diet for 3, 12, 48 and 72 h employing the RNAeasy Plant Mini kit (Qiagen, Germany), which include an on-column DNA digestion step (Qiagen, Germany). Total RNA was quantified with a Nanodrop spectrophotometer (Thermo, USA), and the integrity was assessed on a 1% (w/v) agarose gel. For all samples the obtained RIN was > 7.0, as is needed to follow with the protocol below. Total RNA extraction was do in triplicate for each of the interaction times, i.e., a total of 12 samples were obtained and sent for sequencing (see below).

### Dual RNA-seq and subsequent mapping

High-quality RNA from each sample was sent for RNA sequencing on an Illumina NovaSeq 6000 platform (Macrogen Inc., South Korea). TruSeq Stranded Total RNA with Ribo-Zero H/M/Gold was used to remove both cytoplasmic and mitochondrial rRNA, and cDNA library preparation. About 1.44 × 10^9^ paired-end reads of 200 bp was generated. Raw sequencing reads were trimmed and filtered using Trimmomatic v0.39^21^ with slide window of 3 nt and minimum length of 36 nt; i.e., reads with less than 36 nt were removed from the analysis. Then, the surviving reads were mapped to reference indexes using HISAT 2.1.0^22^. Since the sequence reads come from a mixture of variable proportions of host and pathogen transcriptomes, they need to be sorted into the genomes of corresponding organisms prior to transcriptome assembly and quantification. The available genome assembly used for the host was Tcas5.2 (GenBank Assembly ID: GCF_000002335.3), which corresponds to *T. castaneum* strain Georgia GA2; while the fungal genome assembly used was ASM28067v1 (GenBank Assembly ID: GCF_000280675.1), which corresponds to *B. bassiana* strain ARSEF 2860. Both genomes and annotations files were downloaded from the NCBI Reference Sequence Database^23^.

Although the *B. bassiana* strain used for the experiments is that of the reference genome (ARSEF 2860), the insects used correspond to a local *T. castaneum* strain which has no sequenced genome. The difference between these two strains of beetles can lead to a poor mapping, and consequently to a poor performance on the reads sorting step. For that reason, to assign the reads to the host or to the pathogen genome, all sequenced reads were mapped first to *B. bassiana* genome, and the remaining sequences, which are in much higher proportion, were later mapped to *T. castaneum* genome and analyze as described in more detail below. As the number of reads mapped to the *B. bassiana* genome was very small (~ 0.015%) the biological replicates were pooled. Supplementary Table S1 lists the numbers mapped reads on *B. bassiana* genome for each sample. We evaluated the amount of the fungal-mapped reads that can also be aligned to the insect´s genome, this corresponds to very few reads (e.g., in the pool of samples corresponding to the time point of 72h, only 11 from 91,930 fungal-mapped reads were found mapping with the beetle´s genome). This result guarantees that the mapping order has no influence on the accuracy of expression values.

The reads that were not mapped to *B. bassiana* genome were tried to be mapped to the *T. castaneum* genome. As was described above, the strain of the available genome differs from the strain of the insects used in this study. Thus, the single-nucleotide variants in the reference genome assembly Tcas5.2 were corrected in an iterative manner. To do that, the reads from all samples (biological replicates and time points) were mapped to genome assembly Tcas5.2 with HISAT2 (--score-min L, -4, -0.5, unique mapping). The BAM file is converted to VCF file with bcftools and SNP was called when the Phred-scaled quality score was greater than 5.0, in this case the base found in the *T. castaneum* genome was replaced by the alternate base when there is only one alternative. A total of 719,234 SNPs were detected and used to produce a SNP-corrected version of the Tcas5.2 genome. This procedure was repeated using the new index computed with SNP-corrected genome, 324,838 SNPs were detected in this case. *T. castaneum* genome was corrected again, and the resulting genome was used in next step. The reads from all samples were mapped again to this two-fold corrected genome with HISAT2 and SNP called from the associated VCF file were used to construct the final genome index with hisat2-build with the additional option --snp. This index contains SNPs with more than one alternate base and indels. This iterative process allowed to considerably improve the mapping rate from ~ 40% before the SNP-correction to almost 60% after correction. Supplementary Table S1 lists the number of mapped reads on the corrected *T. castaneum* genome for each sample.

### Data analysis and annotation

Gene expression levels were estimated using Cufflinks v2.2.1 software with default settings^24^. Differential gene expression (DE) analysis for *T. castaneum* transcriptome was performed using Cuffdiff with default settings^24^. Genes with an adjusted *P*-value < 0.05 were assigned as differentially expressed genes (DEGs).

Gene enrichment analyses were performed in the String server (Version: 11.5 https://string-db.org/) and exported to Cytoscape 3.10.0^25^ by using the StringApp plugin^26^. Gene ontology enrichment plots were constructed using EnrichmentMap plugin^27^, using as input all expressed sequences for *B. bassiana*, while only DEGs for *T. castaneum* and using species genomes as background in Cytoscape 3.10.0. Heat maps and volcano plots were constructed using Mathematica (v12.2 Wolfram Inc) scripts. To illustrate the dynamical changes upon the *T. castaneum* transcriptome profile during infection, a dimensional reduction analysis was done following the protocol by Carrea and Diambra^28^. Basically, genes with similar expression levels (co-expressed genes) were grouped by using an agglomerative hierarchical clustering method UPGMA. The agglomerative process is stopped at a given number of clusters considered suitable for our dataset following the Davies-Bouldin index as a measure of the clustering merit. Thus, the temporal expression profile of *T. castaneum* was grouped in 336 clusters. After that, the log-transformed relative expression level of genes within each cluster was averaged to quantify the activity of the clusters at each time point.

### RT-qPCR

Four fungal genes and five insect genes expressed in 48h-interaction samples (see “Results” section) were measured by either two-step (fungi) or one step (insects) reverse transcriptase real-time PCR to validate biologically the RNA-seq assay. The fungal genes selected were those encoding for glucose repressible protein Grg1 (BBA_00807), benzoquinone oxidoreductase (BBA_01593), peptidase inhibitor I9 (BBA_06856), and mannitol dehydrogenase (BBA_06629). The insect genes selected were those encoding for vitellogenin (XM_965117), fatty acid synthase (XM_008202063), lim homeobox 3 (XM_008195361), JNK-interacting protein 1 (XM_962235), and spaetzle 3 (NM_01160153). The relative expression levels were calculated and expressed as the normalized relative quantity (NRQ)^29^ using *B. bassiana* tubulin and *T. castaneum* RPL13 and RP49 as the housekeeping genes. Either the iScript cDNA Synthesis kit and iQ SYBR Green Supermix (Bio-Rad, USA) or iTaq Universal One-Step RT-qPCR (Bio-Rad, USA) were used, and amplifications were performed in an AriaMx Real-time PCR System (Agilent, USA) employing 20 ng reverse transcribed total RNA for each sample. The following amplification program was used: denaturation at 95 °C for 10 min, followed by 40 cycles with 3-segment amplification (30 s at 95 °C for denaturation, 30 s at 61 °C for annealing, and 30 s at 72 °C for polymerase elongation). To confirm that only single products were amplified, a temperature melting step was then performed. Negative controls included samples generated without reverse transcriptase as templates. Reactions containing primer pairs without template were also included as blank controls. The assay was performed in duplicate with each of the three independent biological replicates.

## Results

### Fungal infection

**Figure 1:**
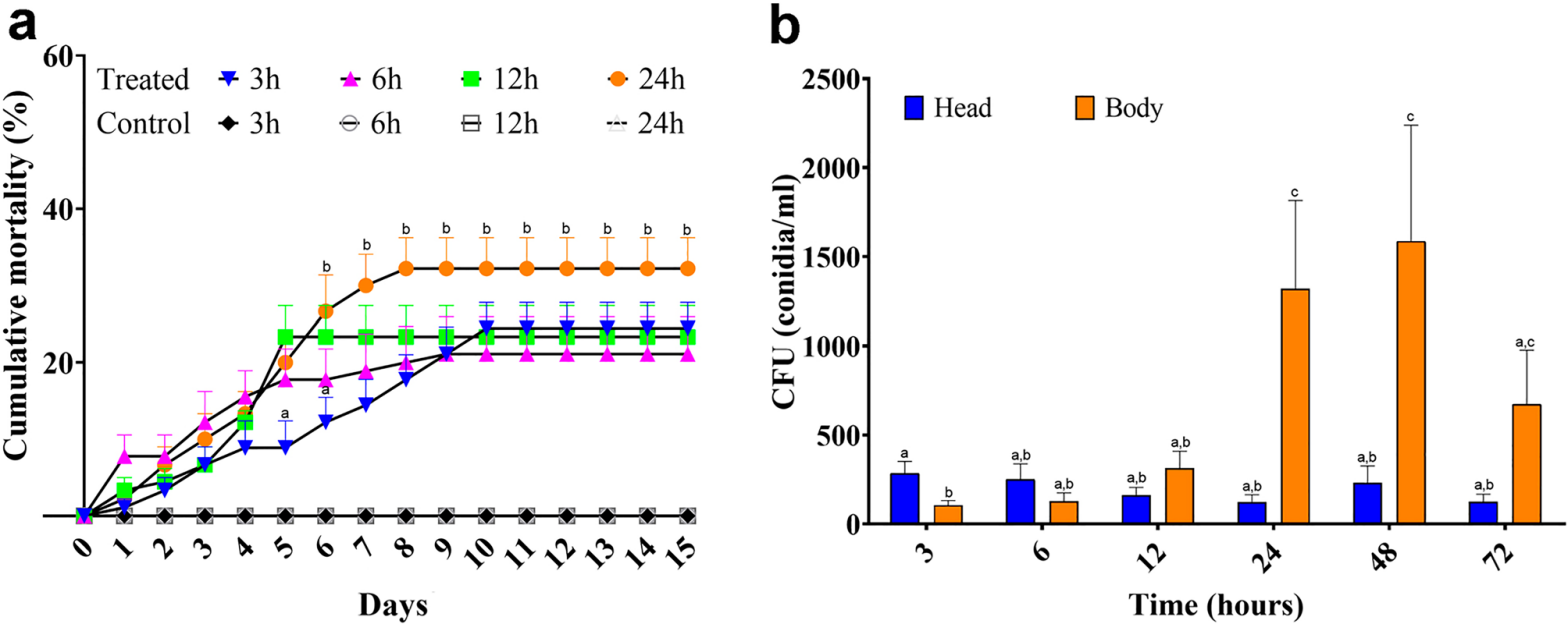
(**a**) Cumulative mortality of *Tribolium castaneum* adults exposed to a mixture of *Beauveria bassiana* conidia for different time periods. Insects were then removed, rinsed to eliminate residual conidia from the surface, and placed on fresh flour. (**b**) Colony forming units (CFU) analysis from internal fungal cells. Adult *T. castaneum* were dissected after interaction with *B. bassiana* conidia for different time periods. Heads and rest of the bodies from previously surface-washed insects (to discard any conidia attached to the surface) were vortexed in Tween 80 (0.01%) and the solution was plated in PDA. In both assays, ten replicates containing ten insects each were used. Bars represent mean ± SD. Different letters indicate significant differences after two-way ANOVA method and Bonferroni’s post test (P < 0.05).

To test a natural way of interaction between adult *T. castaneum* with *B. bassiana* conidia, insects were maintained feeding on conidia-covered corn kernels during different short time periods (i.e., 3, 6, 12, and 24h), and then separated and maintained in rearing conditions. At all time periods assayed, *T. castaneum* started dying around day 2 post treatment, and reached 10% cumulative mortality between days 3 and 4 (Fig. 1a). The maximum cumulative mortality was reached around day 5 post treatment, except for insects fed during 3 and 24h. After 3h-interaction a slower death rate was observed, showing the same mortality than the rest at 6d post treatment, and reaching the higher value after 10d post treatment. For 24 h-interaction, the highest cumulative mortality (30%) was observed after 8d post treatment (Fig. 1a).

Colony forming units (CFU) production is shown in Fig. 1b. Fungal presence appears to reach a maximum in insect heads after 3 h-interaction, since the rest of the time points showed no significant differences between them. The situation is different in the body, where an increase in the presence of conidia was observed at 24 and 48 h, and then slowly decreasing in 72 h-interaction samples (Fig. 1b). This observation indicates that under the interaction protocol assayed, i.e., conidia added to the insect’s diet, the fungal infection starts mostly in the head and then spreads through the rest of the body.

### Dual RNA-seq

To study the gene expression dynamics in both organisms during interaction, a dual RNA-sequencing approach was used. Dual RNA-seq technique enables the simultaneous harvest and monitoring of transcriptional changes during fungal infection, to detect differentially expressed genes during invasion and colonization and, ultimately, to provide a transcriptomic framework for understanding the interactome that emerges during fungus-insect interaction. Total RNA samples from adult insects in contact with conidia during different time periods were collected and subjected to Illumina sequencing, and the resulting paired-end reads were aligned to the genomes of *B. bassiana* and *T. castaneum*. From all samples analyzed (i.e., 3, 12, 48, and 72 h), a total of 1.44 × 10^9^ pair-end reads were obtained; 216,942 pair-end reads were mapped to *B. bassiana* (representing 0.015% of the reads) and 4.03 × 10^8^ were mapped to *T. castaneum* (Supplementary Table S1). In *B. bassiana*, 904 genes were detected in all samples analyzed (Supplementary Table S2). In *T. castaneum*, 14,845 genes were identified in the same samples (Supplementary Table S3).

### Dynamic change of transcripts during the infection process

**Figure 2:**
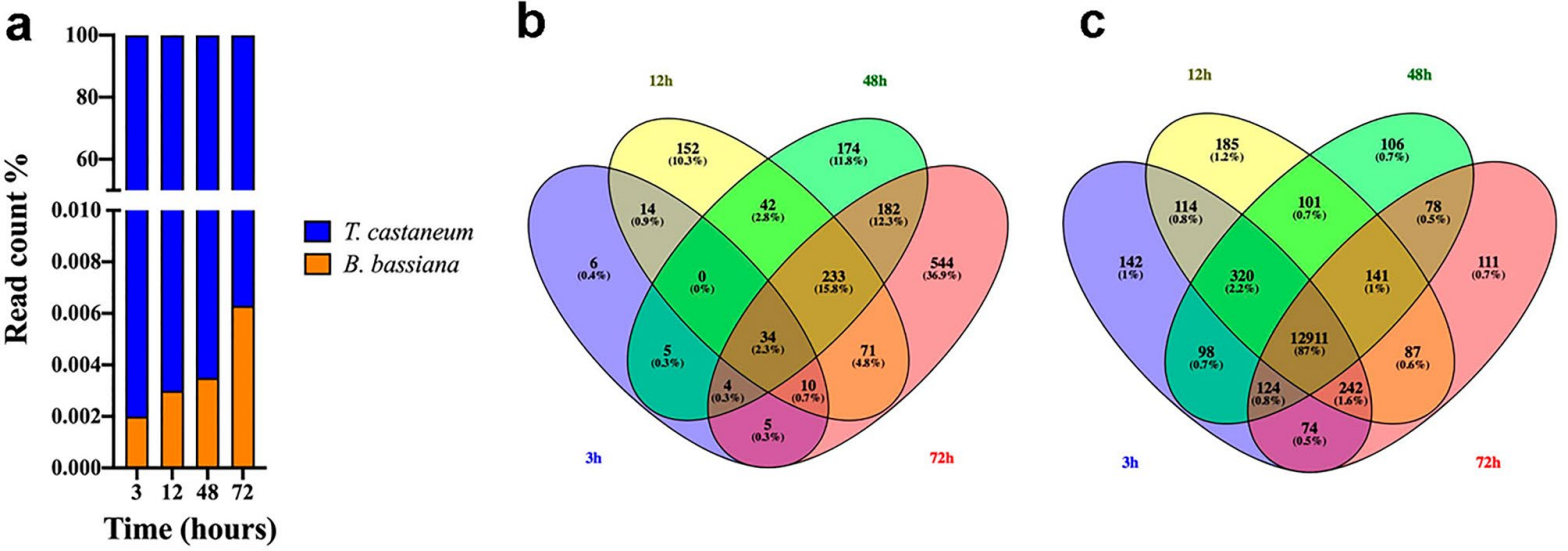
Raw reads analysis. (**a**) Percentage of raw reads corresponding to each organism. (**b**) Venn diagrams represent the amount of reads at each time point for *Beauveria bassiana*. (**c**) Venn diagrams represent the amount of reads at each time point for *Tribolium castaneum*.

To better understand and evaluate the infection progress, it was analyzed how the transcript presence in both organisms varied in time. The proportion of fungal reads relative to beetle reads grew constantly from 3 to 72 h-interaction, increasing from 0.002 to 0.0063%, respectively (Fig. 2a). The Venn diagram (Fig. 2b) showed that fungal transcription is increasing during infection progression (5.2% genes at 3h-interaction, 37.6% at 12 h-, 45.6% at 48 h- and 73.4% fungal genes at 72 h-interaction); also, the percentage of specific fungal genes expressed is progressively increasing at each time point (0.4% at 3 h, 10.3% at 12 h, 11.8% at 48 h and 36.9% at 72 h). In *T. castaneum*, however, most of the genes (87%) are expressed at the four time periods tested (Fig. 2c).

### *B. bassiana*: expression patterns, Gene Ontology (GO) dynamics, and enrichment analysis

Most of the fungal genes expressed within insects at 3 h-interaction are related to nucleosome assembly, ribosomes, chaperons, cell wall, and redox processes (Table 1). In samples from 12 to 72 h-interaction, other high expressed fungal genes were found, primarily related to carbon catabolite repression, cation binding, and peptidase inhibition. A specific benzoquinone oxidoreductase gene (BBA_01593), previously found to interact with tenebrionid secretions^9^, was also expressed within insects at 48 h- and 72 h-interaction (Table 1). To validate the biological conclusions of this part of the study, four fungal genes among the most expressed at 48h-interaction (Table 1) were also measured by RT-qPCR using different biological replicates. BBA_00807 (glucose repressible protein Grg1) was also the most expressed of this set of genes, followed by the mentioned BBA_01593, BBA_06856 (peptidase inhibitor I9), and BBA_06629 (mannitol dehydrogenase) (Supplementary Fig. S1). Genes related to secondary metabolites biosynthesis were identified in 48 h-interaction samples (BBA_03616, encoding for a polyketide synthase from the oosporein gene cluster), and in 72 h-interaction samples (BBA_06727, encoding for a nonribosomal peptide synthase depsipeptides gene cluster) (Supplementary Table S2).

**Table 1:** Top ten ranked genes expressed by *Beauveria bassiana* interacting with *Tribolium castaneum* for different time periods.

Conidia ingestion time	Gene ID	Gene description	FPKM (fragments per kilo base per million mapped reads)
3 h			
	BBA_04053	Choriogenin Hminor	765.270081
	BBA_07091	Conidiation-specific protein 10	623.572205
	BBA_09598	Oxidoreductase2c short-chain dehydrogenase/reductase family	406.896576
	BBA_02383	Hypothetical protein	371.926208
	BBA_00876	Nucleosome assembly protein	352.327393
	BBA_03325	60S acidic ribosomal protein P1	315.497864
	BBA_00176	DnaJ domain-containing protein	299.338715
	BBA_05848	Covalently linked cell wall protein	268.711487
	BBA_03936	DUF1264 domain-containing protein	264.897766
	BBA_04090	Short-chain dehydrogenase	238.594803
12 h			
	BBA_00807	Glucose repressible protein Grg1	4151.953125
	BBA_05166	Hypothetical protein	2071.314697
	BBA_05679	HHE domain-containing protein	2057.088379
	BBA_04088	Hypothetical protein	1753.269531
	BBA_05252	Hydroxyproline-rich glycoprotein DZ-HRGP	1538.462036
	BBA_06856	Peptidase inhibitor I9	1409.003906
	BBA_08893	ATP synthase subunit J	1044.698853
	BBA_06658	60S ribosomal protein L37	1005.352905
	BBA_04720	Hypothetical protein	1001.282349
	BBA_06447	Hypothetical protein	968.785156
48 h			
	BBA_00807	Glucose repressible protein Grg1	6430.260254
	BBA_04088	Hypothetical protein	3220.183838
	BBA_06447	Hypothetical protein	3041.161133
	BBA_04090	Short-chain dehydrogenase	2865.978516
	BBA_06187	Hypothetical protein	2824.542236
	BBA_05166	Hypothetical protein	2649.817627
	BBA_05679	HHE domain-containing protein	2576.352539
	BBA_01593	Quinone oxidoreductase	2274.452148
	BBA_06629	Mannitol dehydrogenase	2208.681152
	BBA_09598	Oxidoreductase 2C short-chain dehydrogenase/reductase family	2150.458008
72 h			
	BBA_05808	Cell wall protein	5790.455078
	BBA_08548	Hypothetical protein	5736.374512
	BBA_00807	Glucose repressible protein Grg1	4440.909668
	BBA_07560	Hypothetical protein	4416.522949
	BBA_02388	Translation elongation factor 1 alpha	3726.454346
	BBA_07138	Conidial wall protein	3092.624268
	BBA_09212	Hypothetical protein	2029.18811
	BBA_03108	Hypothetical protein	1964.206665
	BBA_07240	Hypothetical protein	1879.899048
	BBA_00579	Ubiquitin-conjugating enzyme E2C-binding protein	1752.714355

**Figure 3:**
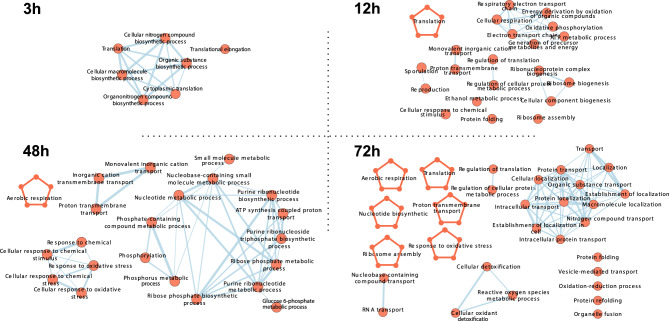
Gene set enrichment analysis derived from all expressed genes of *Beauveria bassiana* within *Tribolium castaneum* at 3 h-,12 h-, 48 h-, and 72 h-interaction. The nodes of the network (orange disks) represent the biological process (BP) terms from Gene Ontology (GO) significantly enriched in the genes expressed at each time point (Bonferroni’s post test, P < 0.05). The blue edges represent gene overlap between gene sets related to different GO terms. Connected nodes are organized in clusters of interconnected BPs obtained by the EnrichmentMap plugin, which considers similarity among gene sets to assign the edges, with a similarity score threshold of 0.5. Orange pentagons indicate collapsed BP clusters to simplify the whole picture. More detailed information about this and other enrichment analyses can be found in the Supplementary Table S4.

As it was mentioned before, the read count for *B. bassiana* in each sample was relatively small; consequently, we decide to pool up the biological replicates. However, this procedure impairs to use of statistical protocols to determine the genes exhibiting differential expression throughout the fungal infection time course. Nonetheless, to extract information from the expressed fungal genes, we conducted an enrichment analysis using all genes identified (Supplementary Table S2), and found different metabolic pathways present as the fungal infection became established. Figure 3 depicts the biological processes (BP) present at each time point. To simplify, some clusters were collapsed to one representative BP, and they were represented by a pentagon. The early expressed genes belong mostly to the translation process (Fig. 3a). From those expressed genes at 12 h-interaction, in addition to BP related to translation, several BPs such as cellular respiration, sporulation and reproduction, biogenesis of cellular components, membrane transport, and protein folding were identified (Fig. 3b). Interestingly, in the later interactions (Fig. 3c, d), several terms associated with the redox metabolism of carbohydrates (glycolysis and the pentose phosphate pathways) and other small molecules that contribute to aerobic respiration, as same as the associated biosynthetic processes, begin to be enriched. For instance, at 48h-interaction several BPs such as those related to response to oxidative and chemical stresses, and nucleotide biosynthesis were enriched (Fig. 3c). Finally, after 72 h-interaction, major enrichment terms corresponded to several BPs related to intracellular transport, cellular detoxification, and response to oxidative stress beside other previously mentioned (Fig. 3d). The enrichment analysis for molecular function (MF), cellular component (CC), and other ontologies is shown in Supplementary Table S4.

### *T. castaneum*: differentially expressed genes (DEGs) and GO enrichment analysis

**Figure 4:**
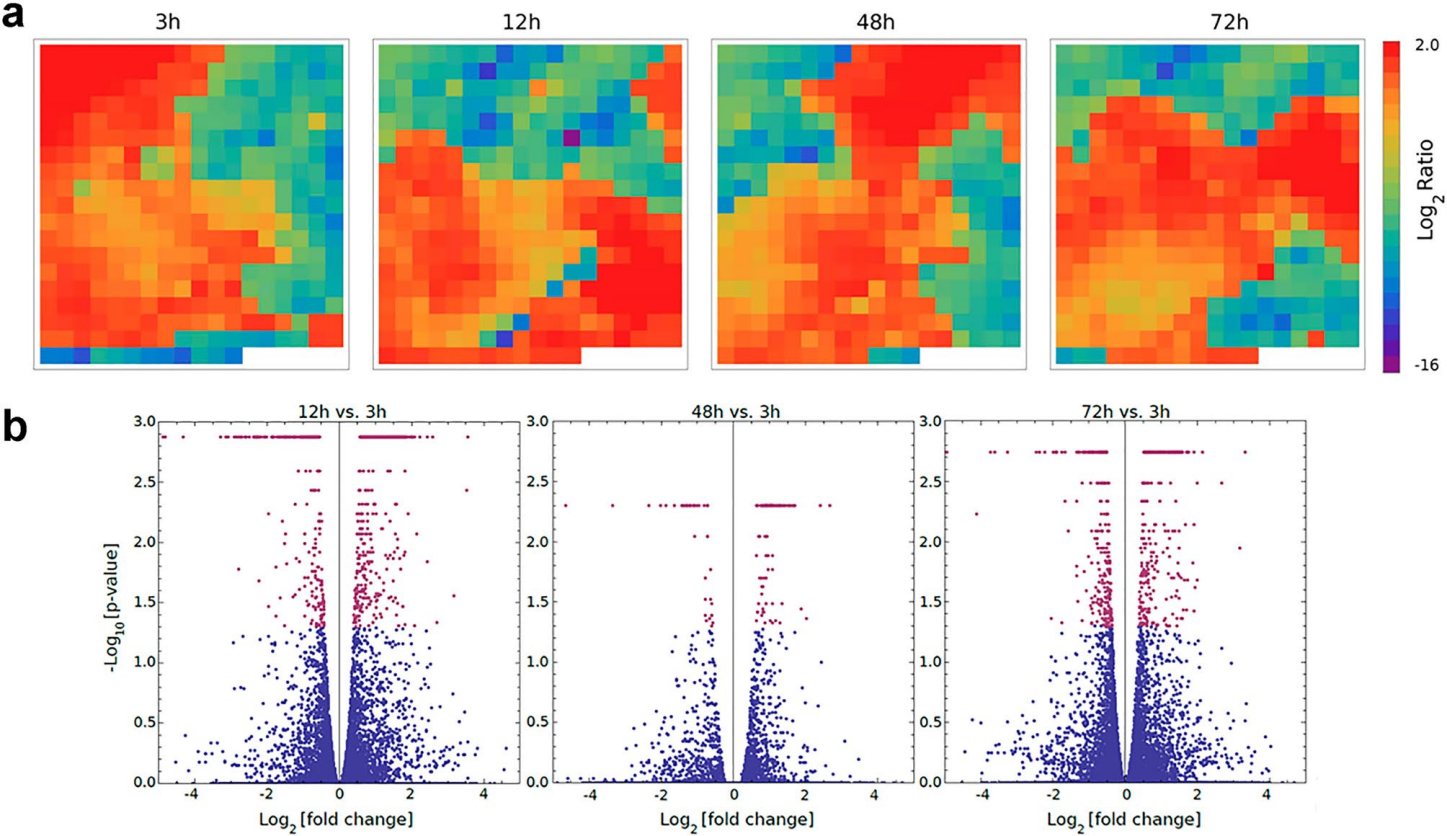
(**a**) Transcriptional snapshots of *Tribolium castaneum* at each surveyed time point. Each cell in the heatmap corresponds to the intra-cluster average of the relative expression level of genes belonging to the cluster. Since position of clusters is the same for all time points, the snapshots can be directly compared among them. (**b**) Volcano scatterplots resulting from the DEGs at 12 h (left), 48 h (center) and 72 h (right), respect to insects at 3 h. As usual, vertical axes correspond to statistical significance (−Log10 (P-value)) and the horizontal axes is the fold change (Log2). Significant genes at 0.05 level (P-value < 0.05) are indicated by red dots.

The expression profile of 14,845 genes of *T. castaneum* is shown in Supplementary Table S3. To validate the biological conclusions from the insect transcriptome, five genes with different expression pattern at 48-h interaction were measured by qPCR. A direct relationship was obtained between the expression values shown by RNA-seq (Supplementary Table S3) and by qPCR (Supplementary Fig. S1). To get a quick visualization of dynamical changes of the host transcriptional response during the infection process, a dimensional reduction analysis was performed. Figure 4a displays a heatmap of the resulting average levels of co-expressed genes after this dimension reduction process. Thus, the *T. castaneum* expression profile was represented by a heatmap of 336 cells, where each cell corresponds to the intragroup average of the activity of the co-expressed genes.

On the other hand, the number of reads obtained for *T. castaneum* allowed to construct the expression profiles through the infective process, and thus DEGs were identified. For insects interacting with conidia for 12 h, 48 h and 72 h, compared to 3h, there were found 284 DEGs (192 up-regulated and 92 down-regulated), 75 DEGs (50 up-regulated and 25 down-regulated) and 190 DEGs (141 up-regulated and 49 down-regulated), respectively (Supplementary Table S5 and Fig. 4b). To explore the significantly lower DEGs obtained for 48 h vs. 3 h interaction, an additional DEG analysis between 12 and 48 h were performed, resulting in 140 DEGs (106 up-regulated and 34 down-regulated) (Supplementary Table S5). After looking the time course for the 24 common DEGs (18 up-regulated and 6 down-regulated) between both comparisons, using 3 h and 12 h as reference against 48 h, a decrease in expression at 12 h can be observed for both up- and down-regulated genes (Supplementary Fig. 5). This means that the differences between 3 and 48 h time points are lower and can lead to a smaller number of DEGs, confirming that this is due exclusively to transcriptomic dynamics.

The main groups of up-regulated genes are linked to cytoskeleton microtubule based mechanisms, transport and cell motility, lipid metabolism mainly linked to Acyl-Coa and lipid transport, aminoacidic metabolism and ubiquitin (Supplementary Fig. S2). Interestingly, two groups of genes related to immune response and stress response were also found. In the immune response cluster, a JNK interacting protein together with pathogenesis-related protein 5—a gene that activates the defense response pathway and enhances the resistance to fungal infection^30^—were included. Stress response cluster included several heath shock proteins encoding genes (Supplementary Table S5 and Fig. S2). Other genes involved in immune signaling were detected as upregulated in early stages; one of them was a LIM factor^31^, together with apoptotic processes related proteins and a Toll-like receptor (Supplementary Table S5 and Fig. S2). Genes involved in DNA and RNA metabolism were highly active, this could be an indicator of a metabolic transition towards immune system activation. Chitinase encoding genes were also identified as over-expressed in early stages. In later stages (48 h-interaction) immune system activation genes such as spaetzle, and stress related genes (pH regulation) were also over-expressed. Interestingly, many genes related to chitin remodeling were found to be up-regulated (chitin synthase, cuticle development and pigmentation). At the last time point analyzed (72 h-interaction), the immune related genes were the most expressed, such as several Toll-receptor like genes, JNK and LIM genes. This expression profile points to a high protein exchange rate throughout the infective process.

For downregulated genes, the gene interaction network analysis showed five clusters linked to development and differentiation, carbohydrates metabolism, chitin metabolism, protein modifications and lipid metabolism, mainly insulin-like peptide, lipase, calcium independent phospholipase encoding genes (Supplementary Fig. S2). As already stated, there are more up-regulated genes than down-regulated genes, and expression patterns generated through the infective process shows a general modification in energetic metabolism and the orchestration of a defensive response.

**Figure 5:**
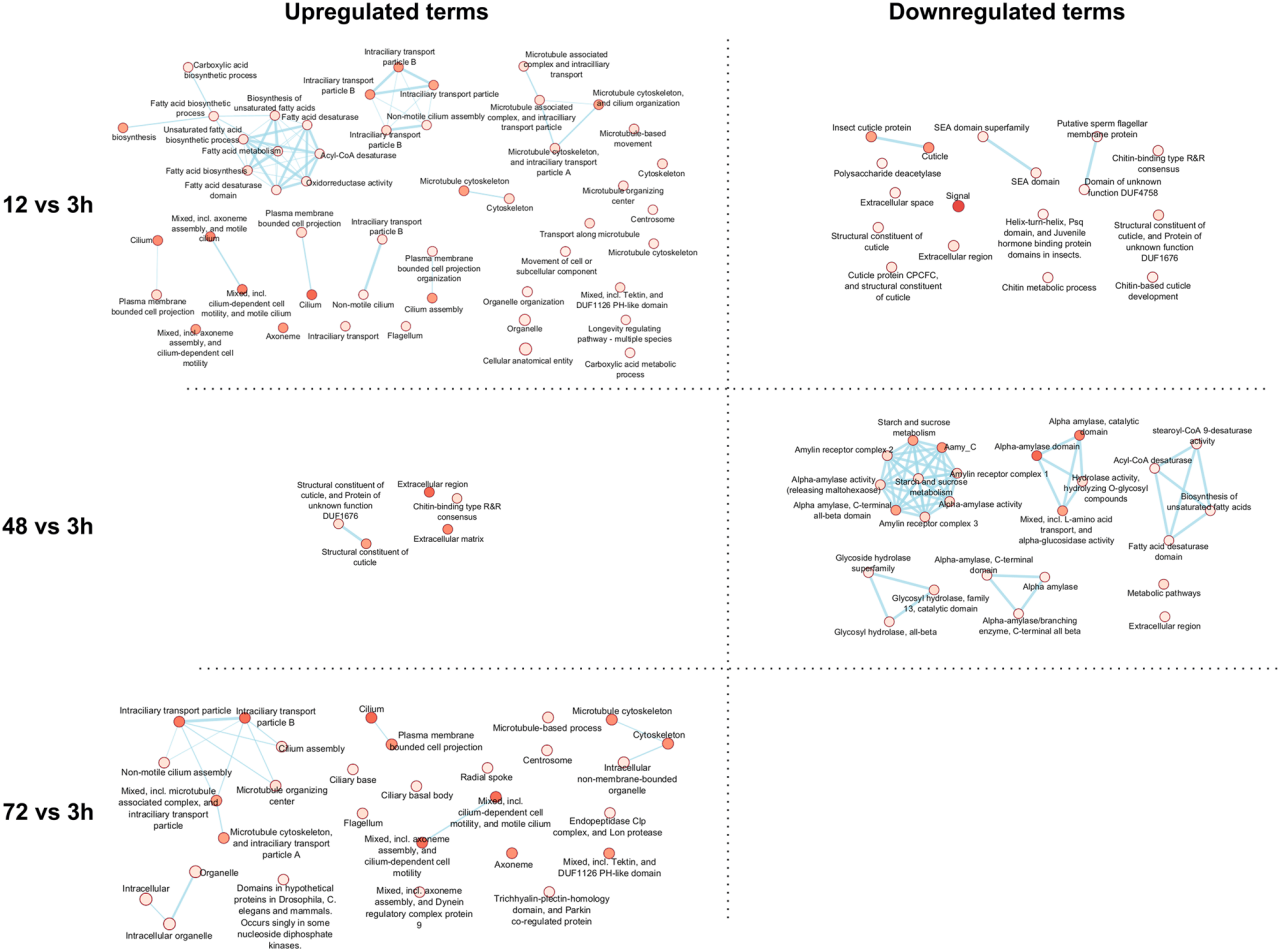
Temporal enriched functional annotations analysis for *Tribolium castaneum*. Functional enrichment based on all DEG up- and down-regulated is represented along a color gradient from light (lower expression) to dark (higher expression) (Cytoscape 3.10.0, EnrichmentMap app) at 12 h-interaction (nine upregulated and three downregulated functional clusters are shown, together with several unclustered enriched annotations), 48 h-interaction (one upregulated and five downregulated annotation clusters are shown), and 72 h-interaction (only upregulated annotation clusters were found).

All DEGs for *T. castaneum* were used for temporal functional GO enrichment analysis in the three categories BP, MF and CC (Supplementary Table S6). At 12 h-interaction, nine clusters of enriched annotations were upregulated compared with 3 h-interaction (Fig. 5a). The main cluster is related to lipid metabolism, specifically to the biosynthetic pathway of unsaturated fatty acids (acyl-CoA desaturase, fatty acid desaturase, oxidoreductase activity), and other carboxylic acid biosynthetic process. The rest of the clusters are mainly linked to transport, i.e., microtubule organization and movement associated to intraciliary transport. Almost all downregulated annotations are related to chitin modifications (Fig. 5a). At 48 h-interaction, few enriched functional annotations linked to the extracellular region and cuticle structure modifications were upregulated (Fig. 5b). Downregulated terms at 48 h-interaction were clustered into five groups, mainly related to sucrose and lipid metabolisms (Fig. 5b). Finally, only upregulated enriched functional annotation was observed at 72 h-interaction, mainly linked to intraciliary transport (Fig. 5c).

## Discussion

Both progression and dynamics of fungal infection through the insect cuticle have been vastly described, the steps that the pathogen takes to infect and colonize the host, and the timing and some of the virulence factors involved in this process are well-known^3,32,33^. Two types of insect immune responses after pathogen infection are also well described: the cellular immunity response that involves phagocytosis, haemocyte aggregation, and pathogen encapsulation; and the activation of humoral immunity that comprises two main immune pathways, IMD and Toll mediated responses. The humoral response culminates with the release of antimicrobial peptides (AMPs) and lectins as a defense mechanism, together with the activation of the prophenol oxidase cascade^34^. *T. castaneum* also has a kind of first barrier composed by an external spray of volatile compounds, mainly benzoquinones, which prevent fungal pathogen attaching to, breaching the cuticle, and infecting the host^3,9^. This strategy confers some tolerance to cuticular infection; therefore, the pathogen must circumvent this hurdle^9^. Within the context of co-evolutionary studies, evidence that *B. bassiana* could infect *T. castaneum* using an alternative route (*per os*) was proposed^35^. Here, we assessed the ability of this entomopathogenic fungus to infect *T. castaneum* when conidia are added to the insect diet, and followed the molecular dynamics by using the dual RNA-seq technique.

Beetles in contact by short time periods with *B. bassiana* conidia added to the diet start to die two days post-contact. As the conidial adhesion process to insect cuticle takes ~ 24 h^3,16^, other mechanism different to cuticle penetration might be being activated. This, together with the fact that *T. castaneum* can prevent conidial adhesion and germination^9^, suggests that *B. bassiana* might be affecting the insect despite having difficulty entering through the cuticle. However, mortalities values obtained with both inoculation techniques, i.e., cuticular infection by insect immersion in conidia suspensions^9^, and a combination of both cuticular and oral infections by conidia added to the insect diet (this study) were quite similar (around 30–40%). This suggest that there could be an intrinsic characteristic in this species leading to little susceptibility to fungal infection, regardless of the inoculation technique or (as a broader conclusion) on the fungus entry mode. On the other hand, we found that at early interaction times fungi can be recovered mainly from the insect head, and 24 h after a higher presence is detected in the rest of the body. A somewhat constant amount of conidia in the head might indicate that *B. bassiana* is not colonizing this region but passing through to the digestive tract. Conidia increase in the body section of insects might correspond to the fungi either colonizing the gut or at least accumulating on it. Once conidia are ingested, they can follow several pathways from blocking the insect respiration^36^ or colonizing the head^37^, to just move through by peristaltic movements and eventually get expelled with the feces^38,39^. Also, it was postulated that *B. bassiana* could disrupt and penetrate the midgut epithelia supported by secretion of secondary metabolites, which allow hyphal bodies growth from the gut^40^. Conidia accumulation into the insect body can be indicative of some of these processes, but more information is needed to trace how fungal cells are distributed within the insect during infection course.

Next generation sequencing platforms are now deep enough and offer the possibility to use dual RNA-seq for simultaneous gene expression assessment from both species involved in the host–pathogen interaction. In this sense, after spraying *Plutella xylostella* larvae with a *B. bassiana* ARSEF 2860 conidial suspension (i.e., cuticular infection), several fungal genes (including biological processes such as antioxidant activity, peroxidase activity and proteolysis) were detected in fungus-infected larvae at 24 h and 48 h post treatment, respectively^16^. Even though the fungal strain was the same as that used in our study, it is very interesting to note that from the more than 200 fungal genes being continuous expressed during infection in *P. xylostella*^16^, only one of them (BBA_07560, hypothetical protein) was found within the almost 40 genes higher expressed inside *T. castaneum*. In other words, the fungus can express different genes during either exclusively cuticular or cuticular/oral infections, and thus the molecular mechanisms involved might be also quite different when infecting different host, by different routes, or both. The different genes detected in both conditions might also be the result of extensive alternative splicing. Dual RNA-seq has been used by Dong et al. to detect this process in *B. bassiana* growing in insect haemocoel^17^. These authors reported that 556 of the 8,840 fungal genes expressed (i.e., 5.4% of the total genes in *B. bassiana*) where subject to alternative splicing.

In our study, the most expressed fungal gene between 12- and 72 h-interaction (BBA_00807) encodes for a putative glucose repressible protein. Carbon catabolite repression factors have been proposed to play important roles in the assimilation of exogenous nutrients and fungal virulence^41^. In this case, nutrient uptake by fungi inside insect might be triggering this gene expression. Also, at 12 h-interaction we found a gene (BBA_06856) encoding for a peptidase inhibitor. Although the mechanisms of fungal peptidase inhibitors are still poorly studied, the inhibitor and pathogen avirulence factor Avr2 from the tomato pathogenic fungus *Cladosporium fulvum* was found to be involved in plant colonization and suppression of host defense^42^. Thus, BBA_06856 expression could be important as defense system protecting *B. bassiana* cells from host defense; specifically, it might avoid degradation by insect peptidases in *T. castaneum* midgut if ingested. Lipid transport and metabolic processes was found also very active in *B. bassiana*, especially linked to biosynthetic routes. It is noteworthy that protein metabolism (both anabolic and catabolic routes) was also found increased, indicating that there is a high rate for protein exchange. These findings coincide with those reported by other authors^16,18^ and may be related to the fact that the pathogen is adapting to invade the host, and thus needs to redirect its working machinery towards infection. Also, redox processes and stress response showed patterns of high sequence count, which suggest that the fungal pathogen is trying to overcome the vast defense system that *T. castaneum* can display.

Regarding host response, the three categories of GO terms (biological process, molecular function, and cellular components) showed a coherent and complementary change of expression pattern among them, displaying expression profiles that show a great change in the metabolic dynamics of chitin structure and energetic metabolism, together with the activation of genes participating in the immune system. In concordance with our results, Chu et al.^16^ also reported more up-regulated than down-regulated genes in fungus-infected insects. These authors found between 2000 and 3000 genes differentially expressed during infection, mostly involved in various immune processes, such as complement and coagulation cascades, protein digestion and absorption^16^. The main immune system regulatory pathways actively crosstalk at the transcription factor level (Dif1 and Rel) in *Drosophila melanogaster*, or their homologues in other insect species^43^. In our case, the RNA-seq results only showed the presence of Toll and Toll-like receptors as well as proteins involved in the JNK pathway, as expected for fungal infections, but no AMPs. This absence might be due to the span of time analyzed since the AMPs are the final and persistent long time effectors of humoral immunity and we are only detecting over expression at the receptor-ligand level (Toll-like-spaetzle). Consistent with the observations by Johnston et al.^44^, we identified a downregulation in many metabolic genes. While they reported repression in glucose metabolism, as well as lipid and vitamin biosynthesis, we also found downregulation in lipid metabolism and the specific suppression of insulin signaling when Toll pathways are activated, as was previously reported in *Drosphila*^45^. Our results support the hypothesis of activation of a general mechanism in which a trade-off between metabolism and immunity takes place^43,45^. This trade-off could be responsible for a change in the transcriptomics dynamic, causing a switch between regular insect metabolism and the onset of the insect immune system to face the infective process.

We were unable to establish any clear correlation between the expressed fungal and insect genes at each time point analyzed. At 48-h interaction, half of the top ten fungal genes expressing non-hypothetical proteins corresponded to the short-medium chain dehydrogenase/reductase family of proteins (BBA_06629, BBA_04090 and BBA_09598), which are known to share significant sequence similarity among members^46^, and to interact with several potential substrates^47^, thus a specific function cannot be assigned. The two most upregulated DEGs in the beetle at 48-h interaction corresponded to cuticle proteins (XM_961452 and XM_965288), even though the fungus does not significantly express proteases or chitinases at this interaction time. Although an insect surface remodeling due to cuticular fungal infection cannot be ruled out, it is also possible that it occurs during an oral infection, since the insect's foregut and hindgut are covered by cuticle^48^. A similar situation was found at 72-h interaction, where UDP-glucuronosyltransferases (catalyzing unspecific glucuronidation of both xenobiotics and endogenous lipophilic compounds) were the three most upregulated DEGs in insects, and two fungal cell wall proteins (BBA_05808 and BBA_07138) represent almost half of the top ten fungal genes expressing non-hypothetical proteins at this late interaction time. Focusing on pathogenesis, cell wall proteins have been proposed as virulence factors in pathogenic filamentous fungi by evading the host immune recognition^49^.

A similar approach (i.e., dual RNA-seq of fungi but infecting only through the insect cuticle) was also performed by Wang et al.^18^ in *Nilaparvata lugens* sprayed with *M. anisopliae* conidia. These authors reported that 2773 and 3895 fungal genes were expressed in the fungus-infected adults at 48 h and 72 h post treatment, respectively, representing about 0.01% of the total reads. In the same fashion to our study, *M. anisopliae*-infected *N. lugens* respond to fungal attack by upregulating most of the ~ 22,000 genes detected, including those involved in host cuticle formation, immune response, and cell detoxification. The mentioned dual RNA-seq studies by Chu et al.^16^ and Wang et al.^18^ reported more fungal genes (~ 2000 to 5000) being expressed inside insects than our study (~ 1000). This is expected since both approaches are based exclusively in cuticular infection process, in which the inoculation method by spraying conidia leads to fungal massive invasion compared with our combined cuticular/oral infection design.

This study represents the first molecular approach to help understand the interaction between an entomopathogenic fungus and its insect host fed a conidia-supplemented diet. Within this infection protocol, it is expected that conidia can potentially enter to the insect by both cuticular and oral routes. However, the contribution of each route to the overall infection cannot be estimated, both the size of adults *T. castaneum* and the way in which they feed mounted on the food source prevent having a "pure" oral infection model. Additional research is needed to complete the entire picture, including histopathological studies to trace the fungal infection dynamics, and the functional characterization of those highly expressed fungal genes identified in this study.

### Supplementary Information

Supplementary Figures.

Supplementary Table S1.

Supplementary Table S2.

Supplementary Table S3.

Supplementary Table S4.

Supplementary Table S5.

Supplementary Table S6.

Supplementary Table S7.

## Data Availability

The dataset generated for this study can be found in the European Nucleotide Archive (ENA) with the accession number PRJEB46827.
