# Supplementary material for: Transcriptomic landscape of the interaction between the entomopathogenic fungus Beauveria bassiana and its tolerant host Tribolium castaneum revealed by dual RNA-seq
Source: Sci Rep. 2023 Oct 2;13:16506. doi: 10.1038/s41598-023-43889-y (PMC10545715; doi:10.1038/s41598-023-43889-y)
Supplement: Supplementary file 1 — Supplementary Figures. [file 41598_2023_43889_MOESM1_ESM.pdf]

Supplementary Figures

Scientific Reports

Transcriptomic landscape of the interaction between the entomopathogenic fungus *Beauveria bassiana* and its tolerant host *Tribolium castaneum* revealed by dual RNA-seq

María Constanza Mannino[1], Belén Davyt-Colo[1], Carla Huarte-Bonnet[1], Luis Diambra[2,3], Nicolás Pedrini[1]

[1]Instituto de Investigaciones Bioquímicas de La Plata (INIBIOLP), CCT La Plata Consejo Nacional de Investigaciones Científicas y Técnicas (CONICET) - Universidad Nacional de La Plata (UNLP), calles 60 y 120, 1900 La Plata, Argentina.

[2]Centro Regional de Estudios Genómicos (CREG), Facultad de Ciencias Exactas, Universidad Nacional de La Plata (UNLP), Boulevard 120 1459, 1900 La Plata, Argentina.

3CONICET, Argentina.

* Corresponding Author: npedrini@med.unlp.edu.ar

a

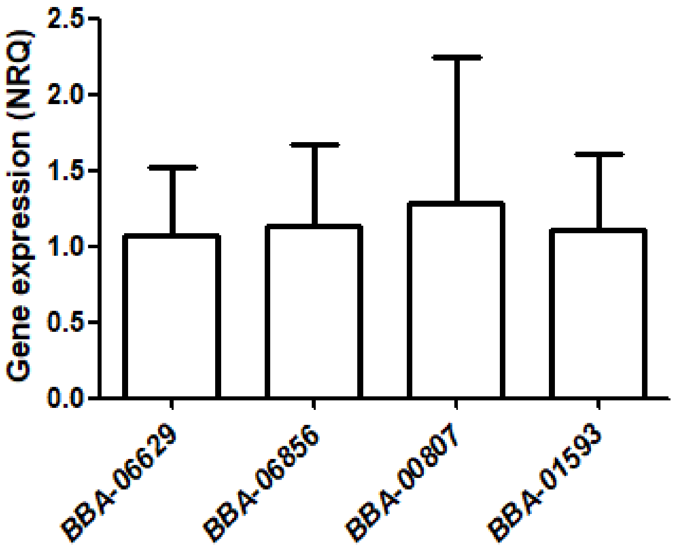

b

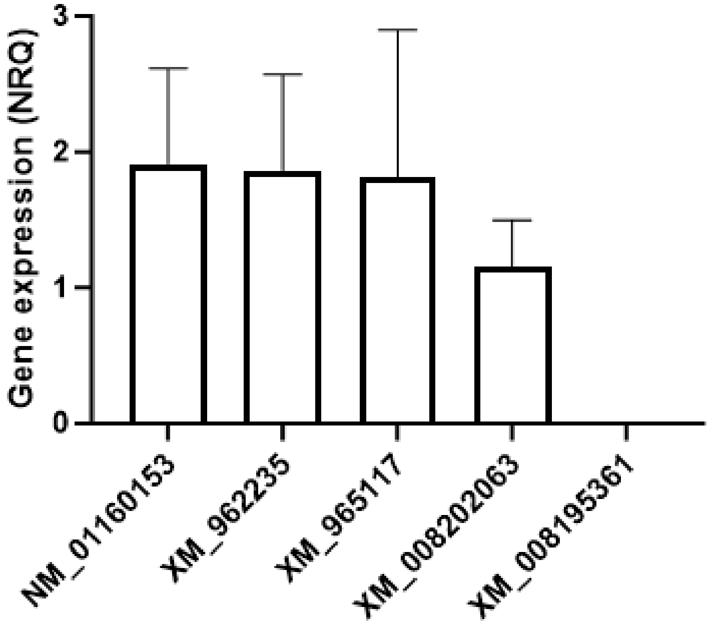

Fig S1: Validation by qPCR of relative expression of four *B. bassiana* genes (a) and five *T. castaneum* genes (b) expressed by RNA-seq at 48h-interaction. Gene expression is showed as normalized relative quantities (NRQ). BBA_06629 (mannitol dehydrogenase), BBA_06856 (peptidase inhibitor I9), BBA_00807 (glucose repressible protein Grg1), and BBA_01593 (benzoquinone oxidoreductase), XM_965117 (vitellogenin), XM_008202063 (fatty acid synthase), XM_008195361 (lim homeobox 3), XM_962235 (JNK-interacting protein 1), and NM_01160153 (spaetzle 3). Primers used are showed in Supplementary Table S7.

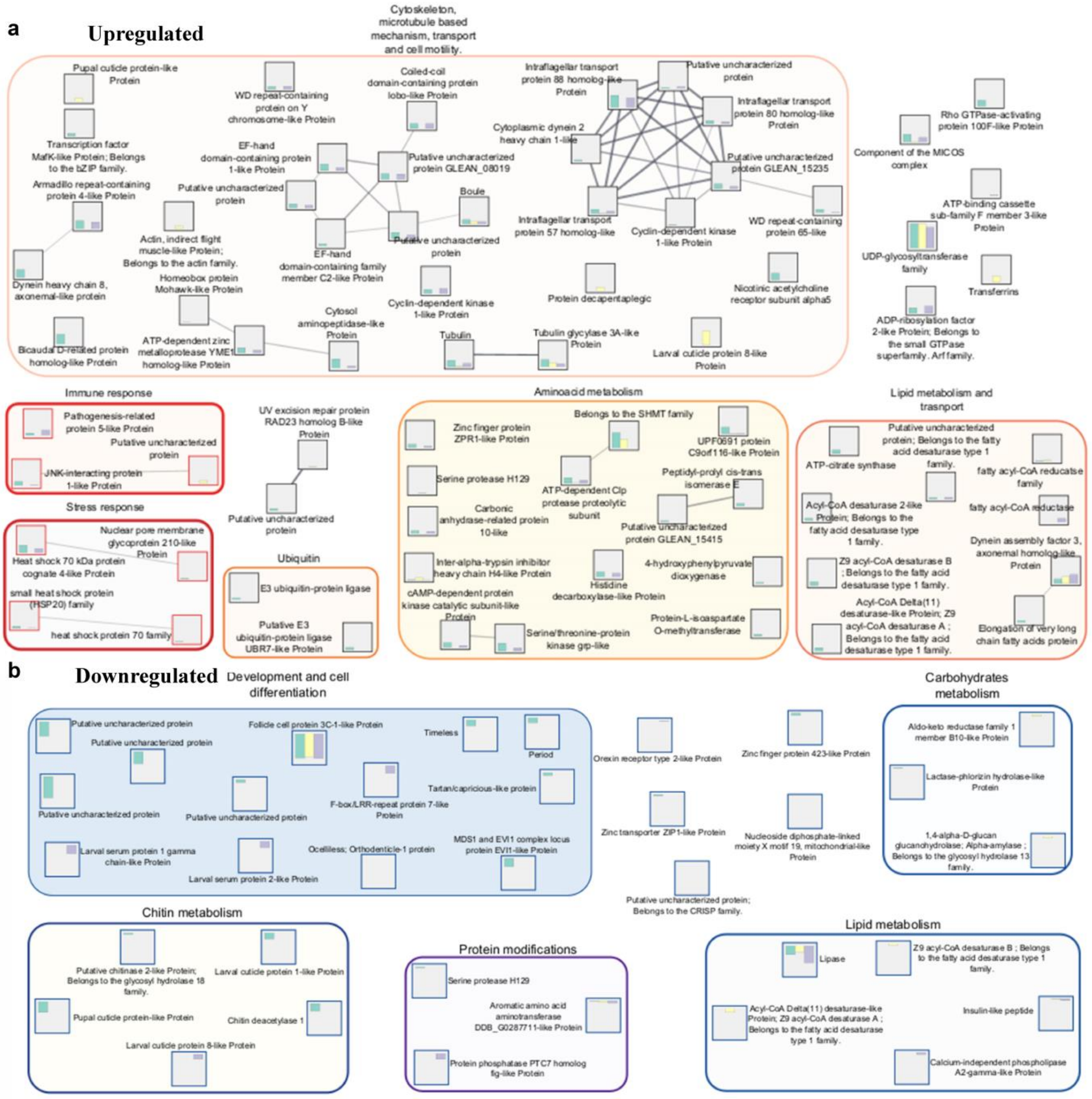

Fig. S2: Gene interaction network in *T. castaneum*. DEG list at all interaction times was subjected to STRING clustering (Cytoscope) and filtered to show the most representative clusters interacting gene products. Bars inside each box indicate expression level at 12h (green), 48h (yellow), and 72h (lilac) compared with 3h.
