# Supplementary material for: Transcriptomic landscape of the interaction between the entomopathogenic fungus Beauveria bassiana and its tolerant host Tribolium castaneum revealed by dual RNA-seq
Source: Sci Rep. 2023 Oct 2;13:16506. doi: 10.1038/s41598-023-43889-y (PMC10545715; doi:10.1038/s41598-023-43889-y)
Supplement: Supplementary file 2 — Supplementary Table S1. [file 41598_2023_43889_MOESM2_ESM.pdf]

Time period	Replicate	Number of paired reads in each sample	Number of paired reads in each time point	Number of reads aligned on Bb concordantly 1 time	subtotal	Number of reads aligned on Tc concordantly 1 time	subtotal
	rep1	58296211		7312		29547506	
3h	rep2	58296811	174893433	7932	29448	28610296	96931792
	rep3	58300411		14204		38773990	
	rep1	62878161		20458		31012726	
12h	rep2	62965211	188613558	16210	44428	34724070	1,05E+08
	rep3	62770186		7760		38938956	
	rep1	65507045		30294		41010208	
48h	rep2	65477635	196463055	9166	51136	40836228	1,15E+08
	rep3	65478375		11676		33119002	
	rep1	54063433		7536		29513424	
72h	rep2	54055413	162205300	9610	91930	27390586	86214920
	rep3	54086454		74784		29310910	

Table S1: Transcriptome sequencing and mapping of *Beauveria bassiana* (Bb) and *Tribolium castaneum* (Tc) interacting during different time periods
