# Supplementary material for: Transcriptomic landscape of the interaction between the entomopathogenic fungus Beauveria bassiana and its tolerant host Tribolium castaneum revealed by dual RNA-seq
Source: Sci Rep. 2023 Oct 2;13:16506. doi: 10.1038/s41598-023-43889-y (PMC10545715; doi:10.1038/s41598-023-43889-y)
Supplement: Supplementary file 8 — Supplementary Table S7. [file 41598_2023_43889_MOESM8_ESM.docx]

**Table S7.** Oligonucleotides used in this study

| **Gene** | **Forward primer (5’ – 3’)** | **Reverse primer (5’ – 3’)** |
| --- | --- | --- |
| *BBA_00807* | GGATTCCATCAAGCAGGGTGTC | GGCCGCTTTCGTTGATCTTG |
| *BBA_06629* | CAAGGGCAAGACGGTCATTGTC | CGCCTTGACCTTGACACCGTAC |
| *BBA_06856* | CAAAGAGGCCAAGGACAAGTGC | TTGGACTCGAGGGTCGTTATGG |
| *BBA_01593* | ACGGCTTCCTGTTTGGCATTCC | GTAATGGATGCCGTGGTGCGTG |
| *BBA_09532** | CATGTTCCGTCGCAAGGCTTTC | CATCATCAATGCCAGCGTCCTG |
| *XM_965117* | AAGGACGCTTCGGTGGAGTCAG | AACAAGCGGCGGTATTCTTTGG |
| *XM_008202063* | CCCGCAACTTAACGACGACTG | GACGCTTGCAGCTGGTGATTC |
| *XM_008195361* | GAGGGCTCCCGTTGATTCAAG | GGATGAAACGGTCGAGGATCAG |
| *XM_962235* | ACTAGGCAGTACAGGGGGAG | GGTTCCTGTGGGAGAATGGG |
| *NM_01160153* | CTGCGCTAAAGTGAGGACGA | TTGCAGTCGTTATCCGGGTC |
| *RPL13** | ACCATATGACCGCAGGAAAC | GGTGAATGGAGCCACTTGTT |
| *RP49** | TGACCGTTATGGCAAACTCA | TAGCATGTGCTTCGTTTTGG |

*** Housekeeping genes
